# Reporting a Patient Safety Culture in Global Health: Evidence From Sierra Leone

**DOI:** 10.1155/jonm/7613998

**Published:** 2025-11-13

**Authors:** Yanran Li, Ning Yang, Lu Niu, Joseph Benjamin Bangura, Mustapha Kabba, Dan Luo, Li Li, Xiang Chen

**Affiliations:** ^1^Xiangya School of Nursing, Central South University, Changsha, Hunan, China; ^2^Clinical Nursing Teaching & Research Section of Xiangya Hospital, Central South University, Changsha, Hunan, China; ^3^Emergency Department of Xiangya Hospital, Central South University, Changsha, Hunan, China; ^4^Xiangya Public Health School, Central South University, Changsha, Hunan, China; ^5^Ministry of Health, Freetown, Western Area, Sierra Leone; ^6^Xiangya Global Health Center, Central South University, Changsha, Hunan, China; ^7^Furong Laboratory, Central South University, Changsha, Hunan, China

**Keywords:** global health, hospital patient safety culture survey 2.0, patient safety culture, Sierra Leone

## Abstract

**Aims:**

To explore the level of patient safety culture in Sierra Leone and identify the factors associated with the patient safety grade and patient safety events reported.

**Design:**

A cross-sectional study.

**Methods:**

From February to March 2024, we selected staff members from three representative public hospitals of different types in Sierra Leone. Patient safety culture was assessed with the Hospital patient safety culture survey 2.0. Binary logistic regression was employed to determine the influence of sociodemographic characteristics on the patient safety grade and patient safety events reported. The textual responses to the open-ended question were imported into Nvivo 12.0 for thematic analysis.

**Results:**

A total of 247 questionnaires was sent out, of which 202 were effectively received. Among 10 safety culture dimensions, three were strength areas with over 75% positive response rate. The other six dimensions ranged from 54.91% to 70.54%. In this study, the highest positive response rate dimension was “Teamwork” (80.83%), and the lowest was “Reporting patient safety event” (49.1%). Item “Staff in this unit work longer hours than is best for patient care”, with a positive response rate of 18.09%, ranks last among all items. “Tenure in Unit/Work Area” could effectively predict patient safety events reported. The initial coding of the open-ended responses yielded a framework of four first-level codes and 11 secondary codes.

**Conclusions:**

It is acknowledged that this is the first research to be conducted in Sierra Leone about the local patient safety culture. Hospital safety culture in Sierra Leone remains suboptimal overall, with adverse event report representing the most significant issue. Our study also uncovers unique patient safety challenges in resource-limited settings (reliable water and electricity supply, proper sanitation, and adequate staffing), addressing a critical gap in the global patient safety evidence base.

**Summary**

• What is already known about this topic?
◦ Economically underdeveloped regions are frequently associated with a higher prevalence of patient safety risks due to systemic healthcare disparities.
◦ Patient safety culture is internationally recognized as a fundamental component for enhancing patient safety and preventing adverse events.
◦ Patient safety culture research is shifting toward developing countries, yet evidence from least developed nations remains critically scarce.
• What this paper adds?
◦ This first patient safety culture survey in Sierra Leone establishes foundational evidence to inform future research, clinical practice, and regional health protection.
◦ Patient safety culture in Sierra Leone falls below average, with “Reporting patient safety event” response positivity ranking lowest across all assessed metrics.
◦ Hospital staff in Sierra Leone most times work beyond safety thresholds, where fatigue and cognitive overload systematically undermine patient safety.
◦ Unique challenges in resource-limited settings were noticed (water/electricity/sanitation), addressing a critical gap in the global patient safety evidence base.
• Patient or public contribution
◦ Nursing administrators from three public hospitals, in collaboration with the China-Africa medical aid team, actively facilitated questionnaire data collection by scheduling interview appointments and providing on-site survey venues.
◦ The participants were involved in the design, or conduct, or reporting, or dissemination plans of our research.

## 1. Introduction

At the latest International Nurses Congress (ICN), patient safety was placed at the top of the agenda, demonstrating its undisputed core priority. There is a significant amount of avoidable patient harm, no matter how advanced the healthcare system [1]. Research shows that 1/10 patients suffers harm during hospital treatment in high-income countries [1], and 13.4 million medical errors occur each year in low- and middle-income countries, resulting in 2.6 million unnecessary deaths [2]. These indirect costs of patient harm amounts to trillions of US dollars each year [3]. A retrospective cohort study shows that adverse events were identified in nearly one in four admissions, and approximately 22.7% events were preventable [4]. Early research efforts concentrated on understanding the extent and typology of human error, which were contributing to patient harm [5]. More recently, the focus of inquiry has shifted towards understanding the role played by human and organizational issues such as leadership, teamwork, and communication in contributing to, as well as preventing, adverse events [6].

Patient safety culture (PSC) was widely recognized as a crucial element in the improvement of patient safety and the prevention of adverse events in healthcare organizations [7, 8], which was sourced from safety culture [9]. Safety culture refers to the beliefs, values, perceptions, and attitudes of patient safety shared among members of the organization and that determined the commitment to, and the style and proficiency of, an organization's health and safety management [10]. PSC is conceptually complex and abstract, which needs interdisciplinary teamwork rather than personal performance and can be viewed within the PSC theoretical framework, which is made up of these components: (a) degree of psychological safety, (b) degree of organizational culture, (c) quality of culture of safety, (d) degree of high reliability organization, (e) degree of deference to expertise, and (f) extent of resilience [11].

Assessing PSC is essential to understand healthcare professionals' perceptions and guide effective interventions. The Hospital Survey on Patient Safety Culture (HSOPSC), developed by the Agency for Healthcare Research and Quality (AHRQ), is one of the most widely used instruments for this purpose in hospitals [12]. Research on PSC has been conducted in various countries to date. A multicenter survey of cancer nurses in four European countries demonstrated that the UK attained the highest patient safety score of 72.0 (17.8), followed by Estonia and the Netherlands, whilst Germany scored the lowest [13]. In a survey of 713 nurses and midwives in Austria, the average score for safety culture that these participants attained was 4.09 ± 0.53 [14]. As patient safety assumes a more prominent role on the global stage, there has been a concomitant shift from developed to developing countries. The mean score achieved by China in relation to its PSC was 72.5 ± 7.6 [15]. A comparative east–west study has revealed that Australian participants reported significantly lower patient safety ratings than their Chinese counterparts [16]. Different demographic characteristics (age, career, work hours, income, educational level, and so on) [17], hospital accreditation [18], work environment [19], interpersonal relationship [20], and communication [21] are all influences on PSC.

It is a matter of concern that PSC is inextricably linked to the regional economy. In some south-east Asian countries, patient safety policy and its application are not prioritized as much as they are in developed countries in the priority [22]. Capnography is universally accepted as an essential patient safety monitor in high-income countries yet is often unavailable in low- and middle-income countries [23]. It is a fundamental right of patients to have access to safe healthcare services, regardless of poverty or wealth. Protecting patient safety in resource-limited settings is an emerging challenge for global healthcare systems [24].

In 2019, AHRQ released a modified HSOPSC, Version 2.0 [25]. Importantly, AHRQ now encourages the use of Version 2.0, and data from the two versions are not directly comparable. Sierra Leone is in the western region of Africa and is one of the least developed countries. Over 60% of people die prematurely before the age of 70 in Sierra Leone, especially women and children, from preventable or treatable causes [26]. A qualitative study revealed a widespread negligence regarding aspects of PSC in African countries, including issues such as material deprivation [27]. There has been limited measurement of PSC in Sierra Leone in previous studies. Thus, this study proposes measuring the level of PSC in Sierra Leone using the latest Version 2.0 and identifying the factors associated with the patient safety grade and patient safety events reported. The findings will fill a gap in previous research and inform administrators and decision makers in promoting a culture of patient safety in Sierra Leone.

## 2. Methods

### 2.1. Participants and Settings

During the initial phase, we have selected 3 representative public hospitals in Sierra Leone by purposive sampling to evaluate the status quo of patient safety. Sierra Leone has few hospitals and relies on international assistance. The hospitals chosen are in Freetown and Makeni, where the Chinese, medical teams work. In Freetown, the capital of Sierra Leone, we selected two public hospitals, which also function as the two most heavily trafficked and representative hospitals. The city of Makeni is home to a single public hospital. Specific information is as follows:

1. Connaught Hospital (principal hospital) (local): Connaught Hospital is Sierra Leone's principal and largest public adult referral hospital. It has staff capacity of 1200 of which 40 are medical doctors (MDs). It comprised of 26 different unites with different specializations, including surgical, radiology, physiotherapy, cancer, epilepsy, mental and psychosocial counseling, accident, and emergency.
2. Sierra Leone–China Friendship Hospital (partnership hospital) (China). It is a partnership hospital. It is a 59-bed capacity hospital providing various services including maternity, surgical, medical, pediatrics, ENT, laboratory, endoscopy daz scan, and x-ray. It has staff strength of 179 of different categories including MDs, lab technicians, pharmacist, anesthetist, and radiographers.
3. Makeni Regional Government Hospital (district and regional hospital) (local): The hospital is the only government referral hospital in Bombali district and the main referral hospital in the northern province. It has a 250-bed capacity, providing primary, secondary, and tertiary services. It comprised of the following technical units: OPDs general, accident and emergency, IPC, mental health, surgical, medical, maternity, pediatrics, SCBU, school health clinic, isolation, pharmaceutical, oral health department, laboratory, ANC, radiology, and ICU.

Finally, hospital staff were recruited from three representative public hospitals by convenience sampling, including physicians, nurses, technicians, administrators, and support staff from February to March 2024. The specific inclusion and exclusion criteria were as follows:

• Inclusion criteria include (1) ≥ 18 years old; (2) participants were legally and formally employed by the respective hospitals; and (3) informed consent.
• Exclusion criteria include (1) absence from work for 6 months or more due to participation in further training, maternity leave, and so on; and (2) unable to understand the questionnaire.

### 2.2. Sample Size Calculation

The minimal sample size was detected by applying a single proportion of the population formula (*N* = *Z*_*α*/2_^2^*P*(1 − *P*)/*δ*^2^) [28], setting *α* = 0.05, *Z*_*α*/2_ = 1.96, and *δ* = 0.1P. We determined the required sample size to be 173, using a 69% response rate derived from the 2022 SOPS Hospital 2.0 Database Report on trending hospitals [29]. Moreover, after adding 20% potential invalid responses, final requested sample size was 207.

### 2.3. Instrumentation

#### 2.3.1. The HSOPSC (2.0 Version).

The HSOPSC 2.0 comprises 40 items, 32 of which are grouped into 10 dimensions which make up the concept of “PSC” [25]. These 32 items are measured on a five-point Likert scale for agreement (from “Strongly disagree” to “Strongly agree”) or frequency (“Never” to “Always”), plus a response option “Does not apply or Don't know.” The instrument also included two single-item outcome measurements, one relating to how often incidents were reported and the other asking respondents to give a patient safety rating in their unit/work area. The six remaining questions request participant data on job position, work unit, length of time working at the hospital and in the present unit/area, weekly hours worked, and whether in direct contact with patients. Percentage of positive responses for each item and dimension was calculated. Responses of “agree” or “strongly agree” and “always” or “most of the time” for the positively worded items indicated positive responses. Additionally, responses of “disagree” or “strongly disagree” and “never” or “rarely” for the negatively worded items indicated positive responses. Positive response rates were used to evaluate attitudes toward PSC in different dimensions. A positive response rate > 75% indicated a strong area of safety culture, while < 50% needed improvement.

Additionally, the survey contained an open-ended item for text entry (please feel free to provide any comments about how things are done or could be done in your hospital that might affect patient safety).

### 2.4. Data Collection

The study was approved and supported by the partner hospitals. As English is the official language of Sierra Leone, the survey was administered in English following an explanation of the purpose, significance, and consent requirements of the study to the hospital authorities and department heads by two members of the research team. Guided by local hospital staff, the research team visited various departments during working hours and invited all available and willing staff to participate in the study. The hospital staff also served as interpreters, as indigenous languages like Mende and Temne were commonly used alongside English. Prior to the survey, all potential participants were fully informed of the study's purpose, procedures, risks, and benefits. Written informed consent was obtained from each individual following a 1-h reflection period with the information sheet. Participants were assured of the voluntary nature of their participation, their right to withdraw at any time without penalty, and the confidentiality of their data. After obtaining informed consent from participants, questionnaires were distributed (paper version). Data collection was carried out by trained researchers from the long-term medical aid team in Sierra Leone. Participants freely chose private settings to complete the questionnaires, and researchers were available throughout to provide immediate clarification for any questions or points of confusion. The completed questionnaires were collected on the same day. All participants demonstrated strong comprehension of English throughout the process.

### 2.5. Data Analysis

Data were analyzed using the IBM Statistical Package for the Social Sciences (Statistics), Version 26.0. Descriptive statistics were estimated to characterize participants by position in this hospital/unit, tenure in hospital//unit, hours worked per week in hospital, and interaction with Patients. Categorical variables were presented as frequencies with percentages. All negatively worded items were reverse coded, and percentage of positive responses for each item and dimension was calculated. Binary logistic regression was employed to determine the influence of sociodemographic characteristics on the patient safety grade and patient safety events reported.

The textual responses from the open-ended question were analyzed using Nvivo 12.0. We conducted a thematic analysis of the responses. The coding process involved an initial review to generate common themes, which were then categorized and refined. After analyzing the suggestions from the first two hospitals, we observed that the responses were encompassed by the existing thematic framework. This consistency in themes was further confirmed by analyzing the responses from the third hospital. Throughout the analysis, we focused on identifying and reporting the themes most relevant to the research topic.

### 2.6. Ethical Approval

The Sierra Leone Ethics and Scientific Review Committee (SLESRC) conducted an expedited review of the above study. The study was conducted in compliance with the latest version of the Declaration of Helsinki. Confidentiality and informed consent were prioritized throughout the investigation. Participants had the option to withdraw from the study at any point with no impact on their work or welfare.

## 3. Results

### 3.1. Participant Characteristics

A total of 247 questionnaires were sent out, of which 202 were effectively received (43 incomplete and 2 invalid questionnaires), giving a response rate of 81.78%. Many of the respondents (58.41%) were nurses, 17.1% from medical/surgical units. Of the respondents, 46.03% had worked in the hospital for 1–5 years, while 27.12% worked for 6–10 years. Half of respondents (50.2%) worked 30–40 h a week. Additionally, 74.8% of respondents had direct contact with patients. See Table 1 for details.

### 3.2. The Positive Response Rate of HSOPSC

Table 2 shows among these 10 safety culture dimensions, three were strength areas with over 75% positive response rate. The other six dimensions ranged from 54.91% to 70.54%. There was one dimension with a positive response rate below 50%, indicating a need for improvement. In this study, the highest positive response rate dimension was “Teamwork” (80.83%) and the lowest was “Reporting patient safety event” (49.1%).

**Figure 1:**
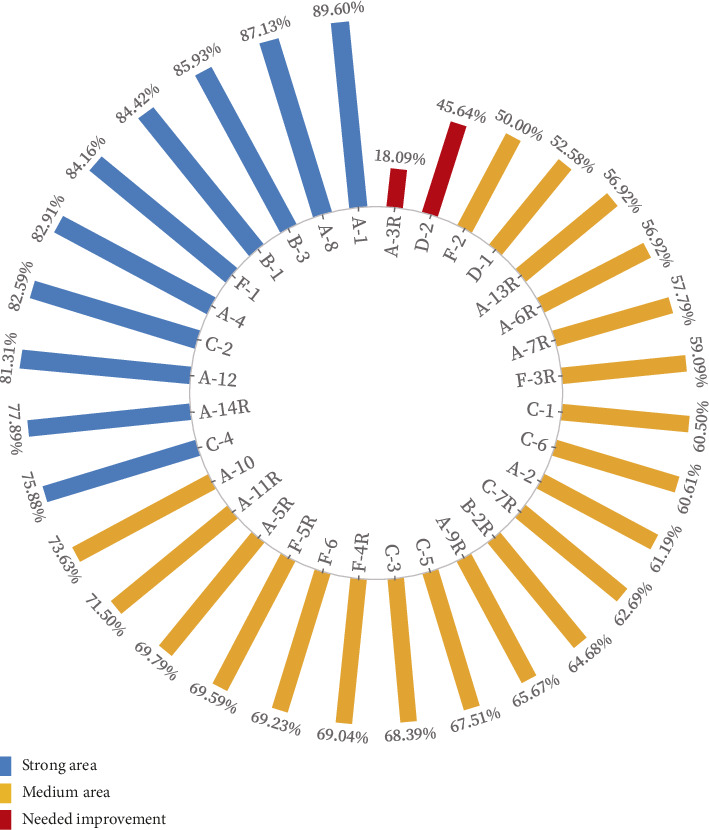
Histogram of patient safety culture in hospitals.

Among the scores of each item of hospital PSC, 10 items are in the strong area, 20 items are in the medium area, and only 2 items are in the needed improvement. Item A-1 (In this unit, we work together as an effective team) has the highest positive response rate, reaching 89.60%. Item A-3R (Staff in this unit work longer hours than is best for patient care) is a reverse item, with a positive response rate of 18.09%, ranking last among all items. Item D-2 (When a mistake reaches the patient and could have harmed the patient, but did not, how often is this reported?) has a positive response rate of 45.64%, only higher than item A-3R. See Figure 1 for details.

### 3.3. Patient Safety Grade and Patient Safety Events Reported

Table 2 shows the majority of participants showed a positive attitude towards their unit, with more than 1/4 rating the overall patient safety rating as excellent. However, in the past year, a whopping 17.33% of patients reported more than 11 patient safety incidents.

### 3.4. Binary Logistic Regression Analysis for Patient Safety Grade and Events Reported

We processed missing data and reclassified demographic variables into binary logistic regression models.  Table 3 shows that “Tenure in Unit/Work Area” could effectively predict patient safety events reported. The probability of employees who had been employed for more than 5 years reporting patient safety events was 2.083 times that of employees who had been employed for less than 5 years, the differences are statistically significant (*B* = 1.13, *p*=0.02). All other results were not significant.

### 3.5. Awaiting Improvement

**Figure 2:**
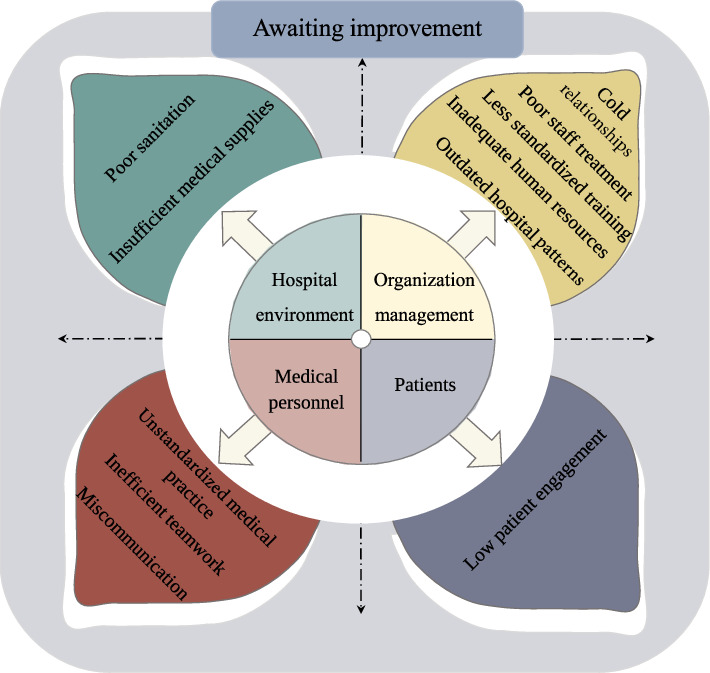
The diagram of awaiting improvement.

The text data came from the last item of the scale survey, and all answers that were clearly written were included in the study. A total of 143 participants filled out the suggestions, of which two phrases whose specific meanings could not be understood were excluded. The suggestions of the remaining 141 participants were included in the final study. Analysis of the open-ended responses revealed a thematic structure comprising four main themes: ① hospital environment aspects, ② organization management aspects, ③ medical personnel aspects, and ④ patients aspects, which branched into eleven subthemes (see Figure 2).

#### 3.5.1. Theme 1: Hospital Environment Aspects

Sierra Leone's hospitals have poor conditions, improper disposal of medical waste, short basic water, electricity and lighting equipment, improper placement of items in the wards, inadequate cleaning, lacking air conditioning and internet, and other problems. The inadequate large quantities of medical supplies, such as ventilators, surgical consumables, masks, and medicines, have brought great challenges to health care services, and even the safety of medical staff themselves is difficult to guarantee.

“Lack of medical equipment (2) Improper disposal of medical waste”

“The hospital management should provide more or enough medical equipment for the hospital so that patients safety would be assured.eg. oxygen cylinder and more.”

“There should be full PPE always…No wheelchair and bed sheets in the wards.”

#### 3.5.2. Theme 2: Organization Management Aspects

At the organizational management level, the nonsatisfactory treatment of medical staff, the limited manpower, limited use of standardized operation guidelines, the outdated hospital model, and the indifferent colleague relationship have brought a series of hidden dangers to patient safety.

“Lack of vital equipment and lack of nurses in the ward”

“(1) Regular Conducting of training facilities for staff (2) Provision of equipment or consumable for continuity or for smooth running of the hospital (3) Conducting regular meetings with staff (4) Regular monitoring on staff (5) no medical for staff”

“The hospital management should have a data Base System that help patient information in the hospital. That can put all the patient card number and time of visit or revisit.”

#### 3.5.3. Theme 3: Medical Personnel Aspects

Poor communication between doctors and nurses will affect teamwork and make it difficult to ensure the safety of patients. In addition, nonstandard medical operations require enhanced awareness and behavioral improvement.

“No proper hand washing”

“We need to work as a team to improve the safety of our patient”

“(5) Poor communication between doctors and nurses”

#### 3.5.4. Theme 4: Patients Aspects

In this topic, little information is obtained, and patient supervision and self-care behaviors need to be added to future medical activities.

“Engage patient in their care plan.”

## 4. Discussion

To the best of our knowledge, this is the first survey on patient safety conducted in Sierra Leone in full response to the call of the AHRQ. These data may facilitate future research and clinical practice related to PSC and safeguard the lives and health of the people in the region.

The overall average positive response rate was 67.62% in our study, which is similar to other low- and middle-income countries (e.g., Iran, 51.32%) and is in the middle of the range [30]. This result is clearly unsatisfactory. It is lower than in some upper-middle-income countries (e.g., Kazakhstan, 73.5%) [31]. “Reporting patient safety event” was the lowest contributing dimension for overall PSC. This means that in Sierra Leone, if a medical worker performs an incorrect medical procedure that does not cause harm to the patient, usually this was not reported. In the regression model, the longer the number of years of service is, the more likely workers were to report adverse events. Combining both, we speculate that there are two reasons: First of all, the reporting behavior of employees is discouraged by a punitive culture [32]. Adverse events trigger a reaction of criticism; younger workers fear this culture and avoid responsibility. The same situation existed in other countries in the past [33], but in recent years, the number of nurses reporting adverse events on their own initiative has gradually increased, thanks to a “nonblame,” communication, and incentives [34]. It is a signal that reporting systems should focus on patient outcomes and learning from systems issues, not blaming individuals. The second is due to irregular report norms, hospital administrators have not made it mandatory for reporting potential adverse events, so young worker do not have the awareness to report [35]. Additionally, even if there was an inclination to report, the irregular report norms would inevitably act as an obstacle. Spontaneous reporting behavior exists among senior staff, as they have more experience related to patient safety and are more aware of the safe practices and benefits of reporting within the hospital [36]. The mentorship is a potential solution to this issue [37]. In international medical assistance aspect, the mentor role (the state that offering relief) not only goes beyond medical tech and services provision but also sets international standards for management. In daily work aspects, the responsibility is shouldered by senior nurses, who are accustomed to providing guidance and support to novice personnel. Furthermore, peer mentoring program is being presented as a viable alternative in consideration of challenging situations [38].

Pay more attention to a phenomenon. Long working hours have become the norm for medical workers in Sierra Leone, which may induce work fatigue and is not conducive to patient safety [39]. Many countries define standard working hours as 35–40 h/week and working ≥ 41 h/week as overtime work [40]; however, about 44% of the participates were working overtime in our study. The shortage of nursing human resources is a common challenge for global health care systems [41]. Whilst other nations can incentivize the migration of nurses via generous remuneration, such expenditures are beyond the financial capacities of African states. The second reason pertains to the act of operating within full manual mode. So far, they lack an electronic database system (mentioned in qualitative information), which means that workers need to spend more time doing some manual and repetitive work. Electronic information systems can assist nurses in timely and standardized nursing operation, yet such systems remain scarce in most low-income countries [42]. There are also major flaws in nursing management. The grassroots nurses are not included in daily nursing management, which will result in nurses no longer feeling a sense of belonging to the hospital. A study revealed that less job embedding represents less innovative behaviors [43]. It is imperative to address the root causes of human resources and manpower management. The reliance on external financial support, such as bailouts, is not a viable long-term solution. The establishment of local medical colleges is a crucial initiative to ensure the effective and sustainable management of these resources. Concurrently, the active involvement and motivation of junior nurses in the management of hospitals may be expected to enhance work efficiency. In addition, in a short conversation with the staff of the hospital, we learned that the hospital would have “volunteers” to assist the regular work in their daily medical activities. In fact, people who do not have the appropriate qualifications are not qualified to practice medicine, which means that the operation of “volunteers” is also one of the risk factors for patient safety.

The analysis of open-ended question highlights a number of existing medical dilemmas, with worrying results. Poor infrastructure such as water, electricity, and lighting, as well as a lack of medical supplies, are the biggest threats to patient safety in Sierra Leone. Obviously, these reality dilemmas are unlikely to change in the short term and no longer a matter of staff awareness. The issue of how to allocate and justify the utilization of healthcare resources has assumed significance in the context of limited resources. Alongside this international assistance, many low- and middle-income countries have established an increasing number of public nongovernmental organization partnerships in the health sector as means of improving public health [44]. In recent years, the medical field has witnessed a paradigm shift towards digital technologies, which has been described as the “digital revolution.” Recent studies have demonstrated the efficacy of mobile health (mHealth) services in addressing issues of scarcity and inequitable distribution of healthcare resources [45]. Daniels noted in his study that the widespread use of geographic information systems for public health issues may be useful for global health outreach planning and resource allocation [46].

A common problem related to hand hygiene. “Unstandardized handwashing” or even “no handwashing” is a patient safety hazard. Standardized training is one way to maintain medical practices and ensure patient safety [47]. User-friendly designs can also promote handwashing; examples include scented hand sanitizers, slogans, dryers, and hand care products.

In summary, the PSC in Sierra Leone is not optimistic, and this requires the contribution and cooperation of various stakeholders. The findings of this study suggest that the state and healthcare organizations should be obliged to implement the following strategies: (1) tolerant workplace culture; (2) mentorship; (3) the establishment of local medical colleges; (4) staff participation in hospital decision-making to build an organizational culture; (5) nongovernmental collaborations; (6) mHealth technology; and (7) standardized training. These strategies are of paramount importance and have the potential to be widely implemented to ensure the delivery of quality care and patient safety.

## 5. Conclusions

It is acknowledged that this is the first research to be conducted in Sierra Leone about the local PSC. Hospital safety culture in Sierra Leone remains suboptimal overall, with adverse event reporting representing the most significant issue. Our study also uncovers unique patient safety challenges in resource-limited settings (reliable water and electricity supply, proper sanitation, and adequate staffing), addressing a critical gap in the global patient safety evidence base.

### 5.1. Limitation

First, a nonprobability sampling approach was employed, which inherently resulted in an unrepresentative sample due to the unequal representation of staff groups, thereby introducing systematic over-representation bias. Second, the questionnaires were self-reported by the participants and the question and answer of the questionnaire items were inaccurate due to factors such as social expectations and memory bias. Finally, due to the scarcity of healthcare resources in the region and the small number of public hospitals and staff, the scope of the survey and sample size need to be expanded in the future.

## Figures and Tables

**Table 1:** Respondent characteristics.

Items	Frequency	Percent (%)
1. Position in this hospital		
Nursing	118	58.41
Medical	15	7.42
Other clinical position	22	10.89
Supervisor, manager, clinical leader, senior leader	3	1.48
Support	11	5.44
Other	8	3.96
Missing data	25	12.37
2. Primary unit/work in this hospital?		
Multiple units, no specific unit	17	8.41
Medical/surgical units	59	29.20
Patient care units	46	22.77
Surgical services	12	5.94
Clinical services	18	8.91
Administration/management	9	4.45
Support services	4	1.98
Other	11	5.44
Missing data	26	12.87
3. Tenure in hospital		
Less than 1 year	47	23.26
1–5 years	93	46.03
6–10 years	44	21.78
11 or more years	17	8.41
Missing data	1	0.49
4. Tenure in unit/work area		
Less than 1 year	74	36.63
1–5 years	101	50.00
6–10 years	20	9.9
11 or more years	7	3.46
5. Hours worked per week in hospital		
Less than 30 h per week	31	15.34
30–40 h per week	82	40.59
More than 40 h per week	89	44.05
6. Interaction with patients		
Yes	176	87.12
No	26	12.87

**Table 2:** The response rate of patient safety culture.

Safety culture	Response rate (%)
Patient safety culture	
Teamwork	80.83
Organizational learning-continuous improvement	80.70
Supervisor manager or clinical leader support for patient safety	78.30
Communication about error	70.54
Hand offs and information exchange	69.28
Communication openness	66.67
Hospital management support for patient safety	64.45
Response to error	61.39
Staffing and work pace	54.91
Reporting patient safety events	49.10
Composite measures average	67.62
Patient safety events reported	
None	30.20
1 to 2	20.79
3 to 5	20.79
6 to 10	10.89
11 or more	12.33
Patient safety grade	
Poor	3.47
Fair	8.91
Good	27.23
Very good	35.15
Excellent	25.25

**Table 3:** Binary logistic regression analysis.

Variables	Patient safety grade				Patient safety events reported			
	*B*	*p*	Exp (*B*)	95% CI	*B*	*p*	Exp (*B*)	95% CI
Position in this hospital	−0.52	0.10	0.59	[0.32, 1.12]	0.51	0.11	1.66	[0.89, 3.01]
Primary unit/work in this hospital	−0.53	0.11	0.59	[0.31, 1.13]	−0.34	0.30	0.71	[0.38, 1.35]
Tenure in hospital	0.10	0.75	1.11	[0.59, 2.09]	−0.34	0.29	0.71	[0.38, 1.33]
Tenure in unit/work area	0.34	0.44	1.41	[0.59, 3.39]	1.13	**0.02**	3.08	[1.21, 7.88]
Hours worked per week in hospital	−0.11	0.71	0.89	[0.49, 1.62]	0.03	0.93	1.03	[0.57, 1.84]
Interaction with patients	0.66	0.17	1.95	[0.76, 4.99]	0.31	0.48	1.37	[0.58, 3.26]

*Note:* Non-nursing = 1; nursing = 2. Nonclinical units (multiple units, no specific unit/administration/management/support services/others) = 1; clinical units (medical/surgical units/patient care units/surgical services/clinical services) = 2. ≤ 5 years = 1; > 5 years = 2. ≤ 40 h per week = 1; > 40 h per week = 2. Yes = 1; No = 2. ≤ 2 times = 1; > 3 times = 2. Well (very good/excellent) = 1; normal (poor/fair/good) = 2. The value of 0.02 is bolded because it is the only *p* value less than 0.05, indicating that ‘Tenure in unit/work area' is a statistically significant predictor of ‘Patient safety events reported.'

## Data Availability

The data that support the findings of this study are available on request from the corresponding author. The data are not publicly available due to privacy or ethical restrictions.

## References

[1] World Health Organization (WHO) (2023). Patient Safety [EB/OL]. https://www.who.int/news-room/fact-sheets/detail/patient-safety.

[2] The LANCET (2019). Patient Safety: Too Little, but Not Too Late. *The Lancet*.

[3] Slawomirski L., Klazinga N. (2020). The Economics of Patient Safety: From Analysis to Action [EB/OL]. http://www.oecd.org/health/health-systems/Economics-of-Patient-Safety-October-2020.pdf.

[4] Bates D. W., Levine D. M., Salmasian H. (2023). The Safety of Inpatient Health Care. *New England Journal of Medicine*.

[5] Wears R. L., Sutcliffe K. M., Rite E. V. (2016). Patient Safety: A Brief but Spirited History (M). https://www.taylorfrancis.com/chapters/edit/10.4324/9781315599700-2/patient-safety-brief-spirited-history-robert-wears-kathleen-sutcliffe-eric-van-rite?context=ub%26refId=f83a3cbe-a91c-4cdb-9f7a-c4702db9c814.

[6] Lee W., Jang I. (2023). Effect of Nurses’ Professionalism, Work Environment, and Communication With Health Professionals on Patient Safety Culture (AHRQ 2.0.): A Cross-Sectional Multicenter Study. *Journal of Nursing Management*.

[7] Wang S. J., Chang Y. C., Hu W. Y., Shih Y. H., Yang C. H. (2022). Improving Patient Safety Culture During the COVID-19 Pandemic in Taiwan. *Frontiers in Public Health*.

[8] Moreno-Leal P., Leal-Costa C., Díaz-Agea J. L., Castaño-Molina M. L. Á., Conesa-Ferrer M. B., De Souza-Oliveira A. C. (2024). Disruptive Behavior and Factors Associated With Patient Safety Climate: A Cross-Sectional Study of Nurses’ and Physicians’ Perceptions. *Journal of Nursing Management*.

[9] O’Donovan R., Ward M., De Brún A., McAuliffe E. (2019). Safety Culture in Health Care Teams: A Narrative Review of the Literature. *Journal of Nursing Management*.

[10] Harton L., Skemp L. (2022). Medical-Surgical Nurse Leaders’ Experiences With Safety Culture: An Inductive Qualitative Descriptive Study. *Journal of Nursing Management*.

[11] Parker D. (2009). Managing Risk in Healthcare: Understanding Your Safety Culture Using the Manchester Patient Safety Framework (MaPSaF). *Journal of Nursing Management*.

[12] Rubin R. (2020). New AHRQ Patient Safety Guidance. *JAMA*.

[13] Sharp L., Rannus K., Olofsson A., Kelly D., Oldenmenger W. H. (2019). Patient Safety Culture Among European Cancer nurses-An Exploratory, Cross-Sectional Survey Comparing Data From Estonia, Germany, Netherlands, and United Kingdom. *Journal of Advanced Nursing*.

[14] Glarcher M., Kaiser K., Kutschar P., Nestler N. (2022). Safety Climate in Hospitals: A Cross-Sectional Study on the Perspectives of Nurses and Midwives. *Journal of Nursing Management*.

[15] He H., Chen X., Tian L. (2023). Perceived Patient Safety Culture and Its Associated Factors Among Clinical Managers of Tertiary Hospitals: A Cross-Sectional Survey. *BMC Nursing*.

[16] Fish J. A., Sharplin G., Wang L., An Y., Fan X., Eckert M. (2022). Cross-Cultural Differences in Nurse Burnout and the Relationship with Patient Safety: An East-West Comparative Study. *Journal of Advanced Nursing*.

[17] Li L. Z., Yang P., Singer S. J., Pfeffer J., Mathur M. B., Shanafelt T. (2024). Nurse Burnout and Patient Safety, Satisfaction, and Quality of Care: A Systematic Review and Meta-Analysis. *JAMA Network Open*.

[18] Oweidat I. A., Atiyeh H., Alosta M. (2024). The Influence of Hospital Accreditation on Nurses’ Perceptions of Patient Safety Culture. *Human Resources for Health*.

[19] Al-Dossary R. N. (2022). The Effects of Nursing Work Environment on Patient Safety in Saudi Arabian Hospitals. *Frontiers of Medicine*.

[20] Migowski E. R., Oliveira Júnior N., Riegel F., Migowski S. A. (2018). Interpersonal Relationships and Safety Culture in Brazilian Health Care Organisations. *Journal of Nursing Management*.

[21] Keshtkar L., Bennett-Weston A., Khan A. S. (2025). Impacts of Communication Type and Quality on Patient Safety Incidents: A Systematic Review. *Annals of Internal Medicine*.

[22] Kang S., Ho T. T. T., Lee N. J. (2021). Comparative Studies on Patient Safety Culture to Strengthen Health Systems Among Southeast Asian Countries. *Frontiers in Public Health*.

[23] Wollner E., Nourian M. M., Booth W. (2020). Impact of Capnography on Patient Safety in High- and Low-Income Settings: A Scoping Review. *British Journal of Anaesthesia*.

[24] Galadanci H. S. (2013). Protecting Patient Safety in Resource-Poor Settings. *Best Practice & Research Clinical Obstetrics & Gynaecology*.

[25] Westat R., Joann Sorra P. D., Naomi Yount P. D., Theresa Famolaro M. P. S., Laura Gray M. P. H. (2019). *Hospital Survey on Patient Safety Culture Version 2.0 User’s Guide*.

[26] Carshon-Marsh R., Aimone A., Ansumana R. (2022). Child, Maternal, and Adult Mortality in Sierra Leone: Nationally Representative Mortality Survey 2018–2020. *Lancet Global Health*.

[27] Aveling E. L., Kayonga Y., Nega A., Dixon-Woods M. (2015). Why is Patient Safety so Hard in Low-Income Countries? A Qualitative Study of Healthcare Workers’ Views in Two African Hospitals. *Globalization and Health*.

[28] Jansen M. S., Groenwold R. H. H., Dekkers O. M. (2024). The Power of Sample Size Calculations. *European Journal of Endocrinology*.

[29] (2023). *SOPS Hospital Database*.

[30] Homaei A., Nemati-Vakilabad R., Ebadi E., Hosseini M., Mirzaei A. (2025). Evaluating Workplace Incivility and Its Relationship With Patient Safety Culture Among EMS Staff: A Cross-Sectional Analytical Study in Iran. *Journal of Nursing Management*.

[31] Aimoldina K., Nurgaliyeva N., Derbissalina G., Vaismoradi M. (2025). Assessing Patient Safety Culture in Nursing Practice in Kazakhstani Healthcare Institutions: A Cross-Sectional Study. *International Nursing Review*.

[32] Feeser V. R., Jackson A. K., Savage N. M. (2021). When Safety Event Reporting is Seen as Punitive. *Annals of Emergency Medicine*.

[33] Logroño K. J., Al-Lenjawi B. A., Singh K., Alomari A. (2023). Assessment of Nurse’s Perceived Just Culture: A Cross-Sectional Study. *BMC Nursing*.

[34] Liu Y., Xu J., Yang X., Yue L., Li G., Mah A. P. (2024). Patient Safety Culture in Private Hospitals in China: A Cross-Sectional Study Using the Revised Hospital Survey on Patient Safety Culture. *Frontiers in Public Health*.

[35] Mayston R., Ebhohimen K., Jacob K. (2020). Measuring what Matters-Information Systems for Management of Chronic Disease in Primary Healthcare Settings in Low and Middle-Income Countries: Challenges and Opportunities. *Epidemiology and Psychiatric Sciences*.

[36] Htet H. Y., Abhicharttibutra K., Wichaikum O. A. (2024). Factors Predicting Proactive Work Behaviors Among Nurses: A Descriptive Predictive Study. *International Nursing Review*.

[37] O’Grady N. (2019). The Role of Mentorship in Trainee Advanced Clinical Practitioner Development. *Journal of Advanced Nursing*.

[38] Jacobsen T. I., Sandsleth M. G., Gonzalez M. T. (2022). Student Nurses’Experiences Participating in a Peer Mentoring Program in Clinical Placement Studies: A Metasynthesis. *Nurse Education in Practice*.

[39] Bae S. H., Fabry D. (2014). Assessing the Relationships Between Nurse Work Hours/Overtimeand Nurse and Patient Outcomes: Systematic Literature Review. *Nursing Outlook*.

[40] Pega F., Náfrádi B., Momen N. C. (2021). Global, Regional, and National Burdens of Ischemic Heart Disease and Stroke Attributable to Exposure to Long Working Hours for 194 Countries, 2000–2016: A Systematic Analysis from the WHO/ILO Joint Estimates of the work-related Burden of Disease and Injury. *Environment International*.

[41] Marć M., Bartosiewicz A., Burzyńska J., Chmiel Z., Januszewicz P. (2019). A Nursing Shortage-A Prospect of Global and Local Policies. *International Nursing Review*.

[42] Witter S., Sheikh K., Schleiff M. (2022). Learning Health Systems in Low-Income and Middle-Income Countries: Exploring Evidence and Expert Insights. *BMJ Global Health*.

[43] Fan S., Zhou S., Ma J., An W., Wang H., Xiao T. (2024). The Role of the Nursing Work Environment, Head Nurse Leadership and Presenteeism in Job Embeddedness Among New Nurses: A Cross-Sectional Multicentre Study. *BMC Nursing*.

[44] Hushie M. (2016). Public-Non-Governmental Organisation Partnerships for Health: An Exploratory Study With Case Studies From Recent Ghanaian Experience. *BMC Public Health*.

[45] Ye Q., Deng Z., Chen Y., Liao J., Li G., Lu Y. (2019). How Resource Scarcity and Accessibility Affect Patients’ Usage of Mobile Health in China: Resource Competition Perspective. *JMIR Mhealth Uhealth*.

[46] Daniels M. J., Game A., Mollura D. J., England R. W. (2021). Strategic Radiology Outreach Planning for Underserved Populations Using Geographic Information Systems. *Journal of the American College of Radiology*.

[47] Tartari E., Fankhauser C., Peters A. (2019). Scenario-Based Simulation Training for the WHO Hand Hygiene Self-Assessment Framework. *Antimicrobial Resistance and Infection Control*.

